# Comparative analysis of soil nematode biodiversity from five different fruit orchards in Osmaneli district, Bilecik, Türkiye

**DOI:** 10.2478/jofnem-2024-0001

**Published:** 2024-03-14

**Authors:** Taylan Çakmak

**Affiliations:** Department of Agricultural Biotechnology, Düzce University Faculty of Agriculture

**Keywords:** community analysis, ecology, nematode diversity

## Abstract

Nematode population densities were determined in 60 soil and root samples collected from 6 fruit orchards in the Bilecik province (western Turkey), between April 2022 and June 2022. The total number of identified nematodes have reached up to 2418 individuals (number of female: 1036; male: 154; and juvenile: 1228). They belong to 54 species, 54 genera, 33 families and 11 orders. Plant parasitic nematodes that were detected mostly are listed as follows: *Helicotylenchus* (6,12 %), *Pratylenchus* (5,74 %), *Paratylenchus* (4.83 %), *Xiphinema* (3,06 %), *Tylenchorhynchus* (2,19 %), *Malenchus* (1.94 %) and *Tylenchus* (1.19 %). According to the maturity index analysis, mean values showed the highest maturity level at peach trees (MI value: 3,52), followed by; walnut trees (MI value: 2.49), cherry trees (MI value: 2.15), nectarine trees (MI value: 1.86), plum trees (MI value: 1.57), and olive trees (MI value: 1.42). Mostly the diverse group in terms of species richness was within the order Dorylaimida. The nematodes associated with peach and walnut trees here showed the most stable environments in terms of soil nematode community structure.

## Introduction

Turkey is in an advantageous position in fruit growing thanks to its location and ecological characteristics. It is known that there are many species and varieties richness in the country due to the fact that it is located in the Near East and Mediterranean gene centers (Erdem and Kos, 2022). The country is the homeland of many fruit species such as that of the apple (*Malus domestica* L.), pear (*Pyrus communis* L.), quince (*Cydonia oblonga* L.), hazelnut (*Corylus avellana* L.), pistachio (*Pistacia vera* L.), sour cherry (*Prunus cerasus* L.), cherry (*Prunus avium* L.), plum (*Prunus domestica* L.), walnut (*Juglans regia* L.), almond (*Prunus dulcis* L.), chestnut (*Castanea sativa* L.), fig (*Ficus carica* L.), grape (*Vitis vinifera* L.) and pomegranate (*Malum granatum* L.), which have an important place in fruit culture. In 2020, a total of 21,853,084 tons of production were realized with an increase of approximately 6% compared to the data of 2019 (Gerçekçioğlu et al., 2009).

The Bilecik Province is located in the southeast of the Marmara Region, between the intersection points of the Marmara, Aegean, Central Anatolia, and Black Sea regions. The region has many advantages in fruit and vegetable cultivation thanks to its location, changing altitude differences in its geography and the unique ecosystem that is surrounded by the Sakarya River. The cultivation of many fruits is well-known locally, such as peach in the Osmaneli district, pomegranate cultivation in the Inhisar district and walnut and cherry cultivation in the Gölpazarı district. Furthermore, Bilecik is at the midpoint of cities such as Istanbul, Bursa, Izmit and Eskisehir, and it also provides access to the Marmara, Central Anatolia and Mediterranean Regions by road and railway, thus providing easy access to airports and ports, and therefore to the market (Niyaz and Demirbaş, 2011) (Table 1).

**Table 1.** Bilecik Province fruit production area and production amount of selected fruit types in 2022.^*^

**Fruit name**	**Production (in tonnes)**	**Total Area of Production(1000m^2^)**	**Yield per tree (kg)**	**Number of matured trees**	**Number of growing trees**
Peach	35.948	20.391	39	911.994	24.38
Cherry	8.209	20.900	28	293.224	124.423
Olive	2.996	16.768	11	269.891	51.83
Walnut	2.157	41.935	18	117.255	130.918
Plum	1.647	3.661	20	81.593	10.521
Nectarine	1	1.700	20	40.01	210
TOTAL	71.024	138.383	2.32	2.154,944	450.346

^*^According to (Erdem & Kos, 2022)

Nematodes (Phylum Nematoda) are one of the most diverse groups of invertebrate animals, characterized by a simple body structure with wide variety of feeding habits, life strategies, and their important role in the soil food web. It is reported that nearly 25.000 nominal species have been identified (Zhang, 2012), and it is estimated that their diversity can reach up to 1.000.000 nematode species (Hugot et al., 2001).

Many species are free-living animals, which inhabit soils and (both freshwater and marine) sediments. Their feeding spectrum is diverse, including predatory, algivory, fungivory, omnivory, saprophagy, etc. (Yeates et al., 1993). Many species have become plant and animal, even human, parasites, causing important diseases and pests (Lee, 2002).

As with any other crop grown in Türkiye, damage to the tree and/or fruit by pests and diseases, including nematodes, reduces the grower’s profit. The effects of some plant parasitic nematodes on plant growth and yield are largely the result of the disruption that these organisms cause to the normal process of plant root growth and soil exploration for both water and nutrients. Nutrient deficiencies resulting from the failure of the plant root system to explore and exploit the soil adequately can also be a major consequence of a plant parasitic nematode attack.

The objectives of this study were, (i) to determine the soil nematode fauna of fruit orchards in Osmaneli District of Bilecik Province, (ii) to characterize of nematodes as soil bioindicators and (iii) to characterize the biodiversity of nematodes in regard to their host plant.

## Material and Methods

This study was established in Duzce University’s Faculty of Agriculture, Department of Agricultural Biotechnology, Nematology Laboratory, from April 2022 to March 2023.

## Sampling

**Figure 1:**
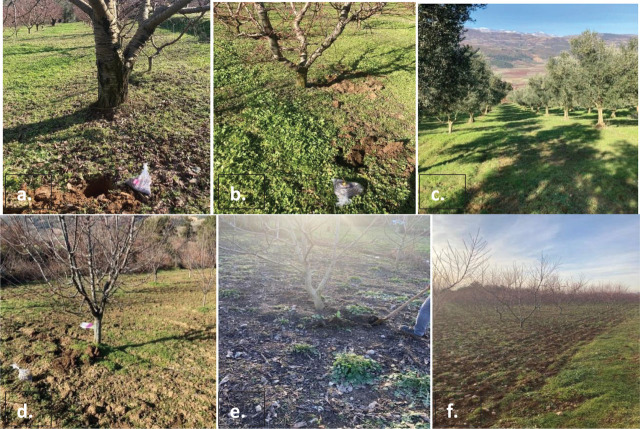
Sampling sites, fruit tree orchards: cherry (a), nectarine (b), olive (c), plum (d), walnut (e), peach (f) trees.

Samples were collected at the Osmaneli district, Bilecik, Turkey, in April 2022 during a field survey. The sampling was done regarding fruit tree orchards soil habitats and along six different eco-habitats, namely: cherry, nectarine, olive, plum, walnut and peach trees. (Fig. 1; Table 2).

**Table 2.** GPS Coordinates of the sampling sites; the location and host plant association.

**N^o^**	**Latitude**	**Longitude**	**Plant/Variety/Local Name**	**Host Plant**	**Location**
1	40.414325	29.847138	Nectarine (Venus®)	*Prunus nectaria* L.	Bereket B. Osmaneli/Bilecik
2	40.414325	29.847138	Nectarine (Venus®)	*Prunus nectaria* L.	Bereket B. Osmaneli/Bilecik
3	40.414325	29.847138	Nectarine (Venus®)	*Prunus nectaria* L.	Bereket B. Osmaneli/Bilecik
4	40.414325	29.847138	Nectarine (Venus®)	*Prunus nectaria* L.	Bereket B. Osmaneli/Bilecik
5	40.414325	29.847138	Nectarine (Venus®)	*Prunus nectaria* L.	Bereket B. Osmaneli/Bilecik
6	40.414325	29.847138	Nectarine (Venus®)	*Prunus nectaria* L.	Bereket B. Osmaneli/Bilecik
7	40.414325	29.847138	Nectarine (Venus®)	*Prunus nectaria* L.	Bereket B. Osmaneli/Bilecik
8	40.414325	29.847138	Nectarine (Venus®)	*Prunus nectaria* L.	Bereket B. Osmaneli/Bilecik
9	40.414325	29.847138	Nectarine (Venus®)	*Prunus nectaria L.*	Bereket B. Osmaneli/Bilecik
10	40.414325	29.847138	Nectarine (Venus®)	*Prunus nectaria L.*	Bereket B. Osmaneli/Bilecik
11	40.414325	29.847138	Cherry (Karabodur)	*Prunus avium* L.	Bereket B. Osmaneli/Bilecik
12	40.414325	29.847138	Cherry (Karabodur)	*Prunus avium* L.	Bereket B. Osmaneli/Bilecik
13	40.414325	29.847138	Cherry (Karabodur)	*Prunus avium* L.	Bereket B. Osmaneli/Bilecik
14	40.414325	29.847138	Cherry (Karabodur)	*Prunus avium* L.	Bereket B. Osmaneli/Bilecik
15	40.414325	29.847138	Cherry (Karabodur)	*Prunus avium* L.	Bereket B. Osmaneli/Bilecik
16	40.414325	29.847138	Cherry (Karabodur)	*Prunus avium* L.	Bereket B. Osmaneli/Bilecik
17	40.414325	29.847138	Cherry (Karabodur)	*Prunus avium* L.	Bereket B. Osmaneli/Bilecik
18	40.414325	29.847138	Cherry (Karabodur)	*Prunus avium* L.	Bereket B. Osmaneli/Bilecik
19	40.414325	29.847138	Cherry (Karabodur)	*Prunus avium* L.	Bereket B. Osmaneli/Bilecik
20	40.414325	29.847138	Cherry (Karabodur)	*Prunus avium* L.	Bereket B. Osmaneli/Bilecik
21	40.416001	29.862371	Plum (Karapapaz)	*Prunus Domestica* L.	Bereket B. Osmaneli/Bilecik
22	40.416001	29.862371	Plum (Karapapaz)	*Prunus Domestica* L.	Bereket B. Osmaneli/Bilecik
23	40.416001	29.862371	Plum (Karapapaz)	*Prunus Domestica* L.	Bereket B. Osmaneli/Bilecik
24	40.416001	29.862371	Plum (Karapapaz)	*Prunus Domestica* L.	Bereket B. Osmaneli/Bilecik
25	40.416001	29.862371	Plum (Karapapaz)	*Prunus Domestica* L.	Bereket B. Osmaneli/Bilecik
26	40.416001	29.862371	Plum (Karapapaz)	*Prunus Domestica* L.	Bereket B. Osmaneli/Bilecik
27	40.416001	29.862371	Plum (Karapapaz)	*Prunus Domestica* L.	Bereket B. Osmaneli/Bilecik
28	40.416001	29.862371	Plum (Karapapaz)	*Prunus Domestica* L.	Bereket B. Osmaneli/Bilecik
29	40.416001	29.862371	Plum (Karapapaz)	*Prunus Domestica* L.	Bereket B. Osmaneli/Bilecik
30	40.416001	29.862371	Plum (Karapapaz)	*Prunus domestica* L.	Bereket B. Osmaneli/Bilecik
31	40.414375	29.869060	Olive (Trilye)	*Olea europaea* L.	Bereket B. Osmaneli/Bilecik
32	40.414375	29.869060	Olive (Trilye)	*Olea europaea* L.	Bereket B. Osmaneli/Bilecik
33	40.414375	29.869060	Olive (Trilye)	*Olea europaea* L.	Bereket B. Osmaneli/Bilecik
34	40.414375	29.869060	Olive (Trilye)	*Olea europaea* L.	Bereket B. Osmaneli/Bilecik
35	40.414375	29.869060	Olive (Trilye)	*Olea europaea* L.	Bereket B. Osmaneli/Bilecik
36	40.414375	29.869060	Olive (Trilye)	*Olea europaea* L.	Bereket B. Osmaneli/Bilecik
37	40.414375	29.869060	Olive (Trilye)	*Olea europaea* L.	Bereket B. Osmaneli/Bilecik
38	40.414375	29.869060	Olive (Trilye)	*Olea europaea* L.	Bereket B. Osmaneli/Bilecik
39	40.414375	29.869060	Olive (Trilye)	*Olea europaea* L.	Bereket B. Osmaneli/Bilecik
40	40.414375	29.869060	Olive (Trilye)	*Olea europaea* L.	Bereket B. Osmaneli/Bilecik
41	40.403494	29.862752	Peach (Cresthaven®)	*Prunus persica* L.	Bereket B. Osmaneli/Bilecik
42	40.403494	29.862752	Peach (Cresthaven®)	*Prunus persica* L.	Bereket B. Osmaneli/Bilecik
43	40.403494	29.862752	Peach (Cresthaven®)	*Prunus persica* L.	Bereket B. Osmaneli/Bilecik
44	40.403494	29.862752	Peach (Cresthaven®)	*Prunus persica* L.	Bereket B. Osmaneli/Bilecik
45	40.403494	29.862752	Peach (Cresthaven®)	*Prunus persica* L.	Bereket B. Osmaneli/Bilecik
46	40.403494	29.862752	Peach (Cresthaven®)	*Prunus persica* L.	Bereket B. Osmaneli/Bilecik
47	40.403494	29.862752	Peach (Cresthaven®)	*Prunus persica* L.	Bereket B. Osmaneli/Bilecik
48	40.403494	29.862752	Peach (Cresthaven®)	*Prunus persica* L.	Bereket B. Osmaneli/Bilecik
49	40.403494	29.862752	Peach (Cresthaven®)	*Prunus persica* L.	Bereket B. Osmaneli/Bilecik
50	40.403494	29.862752	Peach (Cresthaven®)	*Prunus persica* L.	Bereket B. Osmaneli/Bilecik
51	40.402088	29.867829	Walnut (Chandler®)	*Juglans regia* L.	Bereket B. Osmaneli/Bilecik
52	40.402088	29.867829	Walnut (Chandler®)	*Juglans regia* L.	Bereket B. Osmaneli/Bilecik
53	40.402088	29.867829	Walnut (Chandler®)	*Juglans regia* L.	Bereket B. Osmaneli/Bilecik
54	40.402088	29.867829	Walnut (Chandler®)	*Juglans regia* L.	Bereket B. Osmaneli/Bilecik
55	40.402088	29.867829	Walnut (Chandler®)	*Juglans regia* L.	Bereket B. Osmaneli/Bilecik
56	40.402088	29.867829	Walnut (Chandler®)	*Juglans regia* L.	Bereket B. Osmaneli/Bilecik
57	40.402088	29.867829	Walnut (Chandler®)	*Juglans regia* L.	Bereket B. Osmaneli/Bilecik
58	40.402088	29.867829	Walnut (Chandler®)	*Juglans regia* L.	Bereket B. Osmaneli/Bilecik
59	40.402088	29.867829	Walnut (Chandler®)	*Juglans regia* L.	Bereket B. Osmaneli/Bilecik
60	40.402088	29.867829	Walnut (Chandler®)	*Juglans regia* L.	Bereket B. Osmaneli/Bilecik

Samples were collected from 60 sampling sites (10 samples from each fruit type). For each location, one soil sample was collected from a 15 × 15 cm plot. A total number of 60 soil samples were put into ziplock sampling bags, stored in portable cooler during transportation and brought to the nematology laboratory of Duzce University for the extraction process.

## Extraction

A modified Baermann’s (1917) funnel technique using 12 cm diameter petri dishes was used during the extraction of nematodes. After separating rocks, 100 g of fresh soil was evaluated from each sampling site. Plastic trays lined with paper towels were used for extraction and incubated for 48 hours in the nematology laboratory. Extracted nematodes were collected after 48 hours. Nematode suspensions were heated up to 60 °C for killing before fixation. A formalin solution of 4% was used for fixation and preservation of nematodes until preparing permanent glass slides. Extractions were labeled with the relevant sample number, transferred to plastic tubes, and stored at Düzce University Nematology Laboratory. The rest of the soil samples were also stored in the soil laboratory for having a backup requirement in case of future studies.

## Recovery of entomopathogenic nematodes

A 100 g soil sample from each sampling site was placed into a glass container each with three last instar larvae of the wax Moth *Galleria mellonella* (L.) and covered with a lid. (Bedding and Akhurst, 1975; Kaya and Stock, 1997). The samples were then stored at room temperature. After 10 days, dead larvae were collected and transferred to White traps to collect the emerging IJs (Kaya and Stock, 1997).

## Preparation of Nematodes for Light Microscope

After picking up procedure, preserved nematodes were rinsed with purified water to remove the debris. A staining block of 1.25 cm deep which contained 96% ethanol with the extracted nematodes was placed in an incubator at 40 °C, and a few drops of glycerol: formalin (4 %) (1:99) were added and left at room temperature overnight. The next morning, a few drops of a solution of five parts glycerol and 95 parts of 96 % ethanol were added, and two-thirds of its cavity was covered with a glass square. A few drops of the glycerol: ethanol (5:95) solution were added every two hours for the gradual transition of the glycerin. At the end of the day, two drops of glycerol: ethanol (50:50) were added to the staining block. The next day, individual nematodes were covered with glycerol and permanent glass slides were prepared (Yoder et al., 2006).

## Nematode Identification and analysis of ecological parameters

Nematodes were identified manually by using an Olympus CH microscope (Olympus Optical, Tokyo, Japan). Classification of nematodes were determined by a taxonomical key (De Ley and Blaxter, 2005). and additional taxonomical data from Hodda et al. (2006) and Andrássy (2002; 2005; 2009) were included. Nematodes were identified mostly down to the genus level. Coloniser-persister classification of nematode life cycle properties (1–5) were obtained in agreement to Bongers (1990; 1999). The nematode feeding types classification was established according to Yeates et al. (1993) and Du Preez et al. (2022). The structure index and enrichment index were calculated according to Ferris et al. (2001) and Ferris and Bongers (2009) in order to obtain the maturity degree of the nematode community composition in the ecosystem. The Nematode Indicator Joint Analysis calculation system (Sieriebriennikov et al., 2014) was used to analyze food web structure, feeding type diagnostics and MI family indices.

## Results

The total number of identified nematodes reached up to 2418 individuals (number of female: 1036; male: 154; and juvenile: 1228) belonging to 54 species, 54 genera, 33 families and 11 orders (Table 3). Besides, the total nematode abundance showed variability among samples with an average number of nematodes per 100 gr of soil that were 2 to 145 individuals from the sampling sites (Table 2).

**Table 3.** Abundance of nematode genera found on 5 different fruit orchards at Osmaneli, Bilecik, Türkiye

**Genus Name**	**Family Name**	**Walnut**	**Plum**	**Cherry**	**Olive**	**Nectarine**	**Peach**	**C-p class**	**P-p class**	**Feeding type**
*Achromadora* Cobb, 1913	Chromadoridae	9	9	0	0	0	0	3	0	Predators
*Acrobeloides* Cobb, 1924	Cephalobidae	21	54	3	10	0	10	2	0	Bacterivores
*Alaimus* de Man, 1880	Alaimidae	4	0	0	0	0	0	4	0	Bacterivores
*Amplimerliniu*s Siddiqi, 1976	Dolichodoridae	0	0	0	0	4	2	0	3	Herbivores - ectoparasites
*Anatonchus* Cobb, 1916	Anatonchidae	0	0	0	0	2	2	4	0	Predators
*Aphelenchoides* Fischer, 1894	Aphelenchoididae	0	42	5	2	20	0	2	0	Fungivores
*Aphelenchus* Bastian, 1865	Aphelenchidae	19	40	10	0	38	0	2	0	Fungivores
*Aporcelaimellus* Heyns, 1965	Aporcelaimidae	10	6	0	0	0	20	5	0	Predators
*Aporcelaimus* Thorne & Swanger, 1936	Dorylaimidae	12	1	0	0	0	0	5	0	Predators
*Basiria* Siddiqi, 1959	Tylenchidae	4	2	0	0	2	0	0	2	Herbivores - epidermal/root hair feeders
*Belondira* Thorne, 1939	Belondiridae	16	2	0	0	0	2	0	5	Herbivores - ectoparasites
*Belonolaimus* Steiner, 1949	Hoplolaimidae	8	0	0	0	0	0	0	3	Herbivores - ectoparasites
*Cephalobus* Bastian, 1865	Cephalobidae	0	0	0	2	0	0	2	0	Bacterivores
*Cervidellus* Thorne, 1937	Cephalobidae	0	4	0	0	6	0	2	0	Bacterivores
*Chiloplacus* Thorne, 1937	Cephalobidae	11	9	14	8	46	9	2	0	Bacterivores
*Clarkus* Jairajpuri, 1970	Mononchidae	0	0	0	23	0	30	4	0	Predators
*Criconema* Hofmänner & Menzel, 1914	Criconematidae	0	4	0	0	0	0	0	3	Herbivores - ectoparasites
*Diplogaster* Bigot, 1886	Diplogastridae	18	0	0	0	0	0	3	0	Fungivores
*Diphtherophora* de Man, 1880	Diphtherophoridae	0	6	0	5	2	0	1	0	Bacterivores
*Ecumenicus* Thorne, 1974	Qudsianematidae	0	0	0	0	0	10	4	0	Omnivores
*Eucephalobus* Steiner, 1936	Cephalobidae	15	46	4	12	65	0	2	0	Bacterivores
*Eudorylaimus* Andrássy, 1959	Dorylaimidae	0	0	0	2	0	12	4	0	Predators
*Eumonhystera* Andrássy, 1981	Monhysteridae	0	0	13	3	0	0	2	0	Bacterivores
*Filenchus* Andrassy, 1954	Tylenchidae	0	0	1	0	8	0	2	0	Fungivores
*Funaria* Linde, 1938	Leptonchidae	24	0	0	0	0	0	4	0	Fungivores
*Geomonhystera* Andrássy, 1981	Monhysteridae	0	39	0	0	13	0	2	0	Bacterivores
*Helicotylenchus* Steiner, 1945	Hoplolaimidae	2	92	34	10	6	4	0	3	Herbivores - semi-endoparasites
*Heterodera* Schmidt, 1871	Heteroderidae	8	0	0	0	0	0	0	3	Herbivores - sedentary parasites
*Hoplolaimus* von Daday, 1905	Hoplolaimidae	0	0	0	0	1	0	0	3	Herbivores - semi-endoparasites
*Labronema* Thorne, 1939	Dorylaimidae	0	0	1	0	0	0	4	0	Predators
*Malenchus* Andrassy, 1968	Tylenchidae	12	6	14	8	5	2	0	2	Herbivores - epidermal/root hair feeders
*Microdorylaimus* Andrássy, 1986	Qudsianematidae	7	0	0	0	0	0	4	0	Omnivores
*Monhystera* Bastian, 1865	Monhysteridae	14	0	0	0	6	0	2	0	Bacterivores
*Mononchus* Bastian, 1865	Mononchidae	17	0	0	0	0	3	4	0	Predators
*Mylonchulus* Cobb, 1916	Mylonchulidae	0	0	3	13	0	0	4	0	Predators
*Nagelus* Thorne & Malek, 1968	Telotylenchidae	0	14	0	0	0	1	0	3	Herbivores - ectoparasites
*Nygolaimus* Cobb, 1913	Nygolaimidae	0	0	0	0	0	2	5	0	Predators
*Panagrolaimus* Fuchs, 1930	Panagrolaimidae	28	23	0	0	37	11	1	0	Bacterivores
*Paratylenchus* Filipjev, 1936	Criconematidae	23	9	0	0	77	8	0	2	Herbivores - ectoparasites
*Plectus* Bastian, 1865	Plectidae	24	6	0	0	2	0	2	0	Bacterivores
*Pratylenchus* Filipjev, 1936	Hoplolaimidae	28	5	0	101	1	4	0	3	Herbivores - migratory endoparasites
*Prionchulus* (Cobb, 1916) Wu & Hoeppli, 1929	Mononchidae	0	2	0	6	0	0	4	0	Predators
*Prismatolaimus* Micoletzky, 1922	Prismatolaimidae	0	12	0	0	0	0	3	0	Bacterivores
*Rhabditis* Dujardin, 1845	Rhabditidae	16	10	0	0	4	2	1	0	Bacterivores
*Rotylenchus* Filipjev, 1934	Hoplolaimidae	0	0	0	0	8	0	0	3	Herbivores - semi-endoparasites
*Steinernema* Travassos, 1927	Steinernematidae	0	271	0	387	0	0	1	0	Bacterivores
*Telotylenchus* Siddiqi, 1960	Tylenchidae	0	0	0	0	2	0	0	3	Herbivores - ectoparasites
*Teratocephalu*s De Man, 1876	Teratocephalidae	0	0	0	0	1	0	3	0	Bacterivores
*Tobrilus* De Man, 1879	Tobrilidae	0	1	0	0	0	0	3	0	Predators
*Tripylella* Brzeski & Winiszewska-Slipinska, 1993	Tripylidae	9	0	0	0	0	0	3	0	Predators
*Tylenchorhynchus* Cobb, 1913	Belonolaimidae	6	7	15	1	19	5	0	3	Herbivores - ectoparasites
*Tylenchus* Bastian, 1865	Tylenchidae	0	0	2	1	23	3	0	2	Herbivores - epidermal/root hair feeders
*Tylocephalus* Crossman, 1933	Plectidae	0	4	0	0	0	0	2	0	Bacterivores
*Xiphinema* Cobb, 1913	Longidoridae	0	3	56	6	9	0	0	5	Herbivores - ectoparasites

*TOTAL ABUNDANCE*		365	729	175	600	407	142			2418

## Maturity Index Analysis

**Figure 2:**
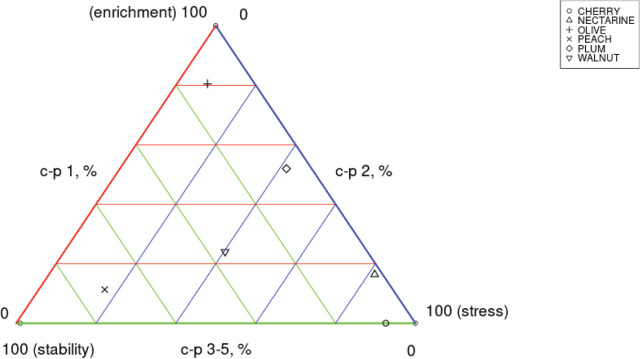
Comparative maturity index analysis of nematode c-p classification from five fruit orchards in Osmaneli, Bilecik, Türkiye.

**Figure 3:**
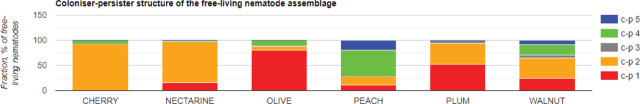
Free-living nematode c-p classification from five fruit orchards in Osmaneli, Bilecik, Türkiye.

According to the maturity index analysis (Fig. 2; 3), mean values showed the highest maturity level at peach trees (MI value: 3,52), followed by; walnut trees (MI value: 2.49), cherry trees (MI value: 2.15), nectarine trees (MI value: 1.86), plum trees (MI value: 1.57) and olive trees (MI value: 1.42). According to the maturity index 2–5 analysis, mean values showed the highest maturity level at peach trees (MI value: 3,84), followed by; olive trees (MI value: 3.16), walnut trees (MI value: 2.96), plum trees (MI value: 2.19), cherry trees (MI value: 2.15) and nectarine trees (MI value: 2.03). According to the Sigma Maturity Index analysis, mean values showed slightly different values: highest maturity level was detected at peach trees (Sigma MI value: 3.35), followed by; cherry trees (Sigma MI value: 3.29), walnut trees (Sigma MI value: 2.62), nectarine trees (Sigma MI value: 2.08), plum trees (Sigma MI value: 1.84) and olive trees (Sigma MI value: 1.76). The plant parasitic nematode index analysis showed the highest PPI mean values at cherry trees (PPI value: 3.79), followed by; olive trees (PPI value: 3.02), plum trees (PPI value: 2.95), walnut trees (PPI value: 2.93), peach trees (PPI value: 2.71) and nectarine trees (PPI value: 2.43). The enrichment index analysis (EI), results showed the highest enrichment level at olive trees (EI value: 97.67), followed by; plum trees (EI value: 84.18), peach trees (EI value: 73.24), walnut trees (EI value: 71.97), nectarine trees (EI value: 53) and cherry trees (EI value: 24.24). According to the structure index analysis (SI), results showed the highest structure level at peach trees (SI value: 95.26), followed by; olive trees (SI value: 85.56), walnut trees (SI value: 78.78), plum trees (SI value: 31.99), cherry trees (SI value: 24.24) and nectarine trees (SI value: 6.74).

**Figure 4:**
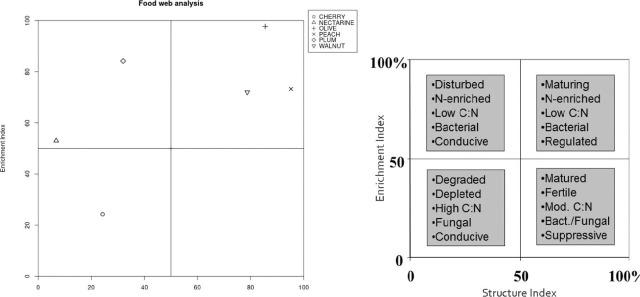
Food web analysis (Enrichment/Structure indices) from five fruit orchards in Osmaneli, Bilecik, Türkiye.

Food web analysis of soil properties provides a useful tool for predicting soil quality by enrichment and structure type parameters. Results showed that the two of the fruit orchards, nectarine and plum, nematode assemblage were yielded into a high enrichment class, which means disturbed, N. enriched, with low C:N value, high bacterial activity and conductive soil. Three of the fruit trees’ (Olive, walnut and peach) nematode assemblage were placed into maturing, N-enriched, with low C:N value, high bacterial activity, and regulated class. Nematode assemblage of the cherry tree orchard occurred at degraded, depleted, with high C:N value, more fungal activity and a conductive soil type (Fig. 4).

## Feeding type composition of nematode assemblage according to tree types

**Figure 5:**
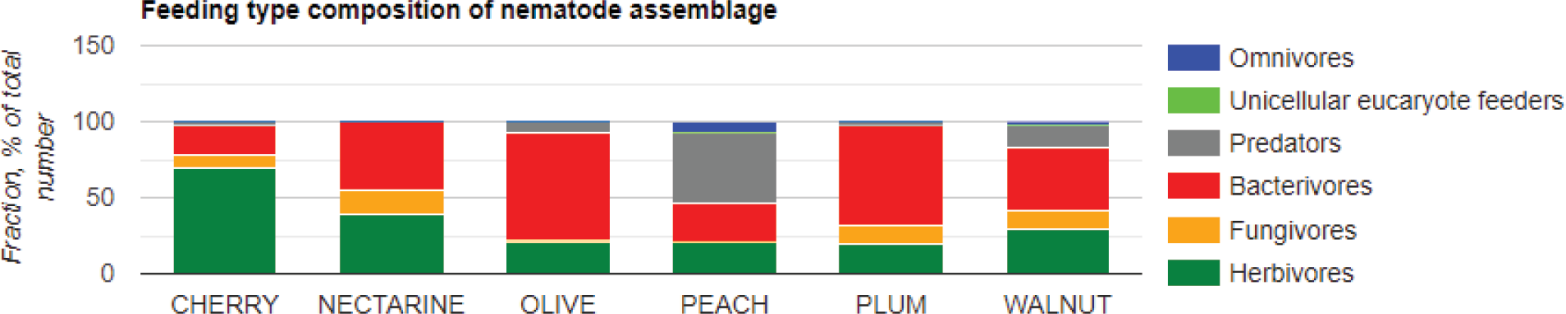
Feeding types and their relative abundance of nematodes at different fruit tree orchards.

According to results obtained by feeding types of cherry trees’ nematode assemblage, plant parasitic nematodes were found to be 69.1 %, followed by bacterivorous nematodes (19.4 %), fungivorous nematodes (9.1 %) and predator nematodes (2.3 %); nectarine trees: Bacterivorous nematodes (44.2 %), plant parasitic nematodes (38.6 %), fungivorous nematodes (16.7 %) and predator nematodes (0.5 %); olive trees: Bacterivorous nematodes (70.7 %), plant parasitic nematodes (20.9 %), predator nematodes (7.2 %) and fungivorous nematodes (7.2 %); peach trees: Predator nematodes (47.3 %), bacterivorous nematodes (24.7 %), plant parasitic nematodes (21.2 %) and omnivorous nematodes (6.8 %); plum trees: Bacterivorous nematodes (65.6 %), plant parasitic nematodes (19.8 %), fungivorous nematodes (12.1 %) and predator nematodes (2.6 %); walnut trees: Bacterivorous nematodes (41.4 %), plant parasitic nematodes (29.3 %), predator nematodes (15.6 %), fungivorous nematodes (11.8 %) and omnivorous nematodes (1.9 %) (Fig. 5).

**Figure 6:**
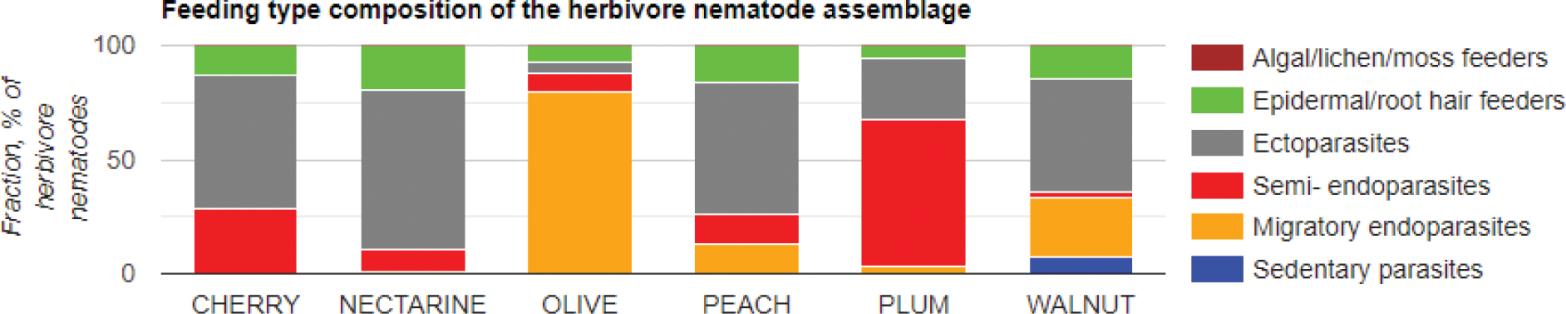
Distribution (%) of feeding types within the plant-parasitic nematodes from Osmaneli, Bilecik, Türkiye.

According to results obtained by the classification of plant parasitic nematode feeding type differences, nematode assemblage in cherry tree orchards were: Ectoparasites (58.7 %), semi-endoparasites (28.1 %) and epidermal/root hair feeders (13.2 %); nectarine trees: Ectoparasites (70.7 %), epidermal/root hair feeders (19.1 %), semi-endoparasites (9.6 %) and migratory endoparasites (0.6 %); olive trees: Migratory endoparasites (79.5 %), semi-endoparasites (7.9 %), epidermal/root hair feeders (7.1 %), ectoparasites (5.5 %); peach trees: Ectoparasites (58.1 %), epidermal/root hair feeders (16.1 %), migratory endoparasites (12.9 %) and semi-endoparasites (12.9 %); plum trees: Semi-endoparasites (63.9 %), ectoparasites (27.1 %), epidermal/root hair feeders (5.6 %) and migratory endoparasites (3.5 %); walnut trees: Ectoparasites (49.5 %), migratory endoparasites (26.2 %), epidermal/root hair feeders (15 %), sedentary endoparasites (7.5 %) and semi-endoparasites (1.9 %) (Fig. 6).

**Figure 7:**
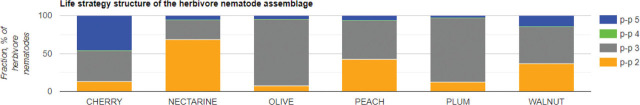
Plant parasitic nematode c-p classification from five fruit orchards in Osmaneli, Bilecik, Türkiye.

According to results obtained by the classification of plant parasitic nematode cp class differences, nematode assemblage in cherry tree orchards were PP5 (46.3 %), PP3 (40.5 %), and PP2 (13.2 %); nectarine trees: PP2 (68.2 %), PP3 (26.1 %) and PP5 (5.7 %); olive trees: PP3 (88.2 %), PP2 (7.1 %) and PP5 (4.7 %); peach trees: PP3 (51.6 %), PP2 (41.9 %) and PP5 (6.5 %); plum trees: PP3 (84.7 %), PP2 (11.8 %) and PP5 (3.5 %); walnut trees: PP3 (48.6 %), PP2 (36.4 %) and PP5 (15 %) (Fig. 7).

## Discussion

### Faunistics

The Bilecik Province, located in the Marmara Region of Turkey, is a region where agricultural production is intense. In this sense, when we look at the structure of the fruit trees in the region, it is possible to say that stone fruits are generally grown. However, fruits such as olives and walnuts are widely grown in the region. In the specific Osmaneli district of the Bilecik province, no holistic study on soil nematode diversity was found in this region in the literature research. However, this climate zone, which is fed with rich water resources with its proximity to the Sakarya river, is a region rich in terms of highly fertile soil structure and therefore biodiversity. Among the studies, records from Turkey were presented in the study of plant parasitic nematode fauna in olive trees in the Mediterranean climate zone (Ali et al., 2014). When the studies on soil nematode fauna from the Marmara Region ( Öztürk, 2023) were examined, it was determined that there were similar taxa. Likewise, a large-scale survey study was conducted mainly on entomopathogenic nematodes (Güneş and Gozel, 2011) from orchards in the Marmara Region.

The rising interest of learning about nematode community behavior in the soil food web is allowing us to monitor their role in the ecosystem. In Türkiye, the discovery of soil nematodes and especially the diversity of free-living nematodes are still receiving very little interest from the scientific community. The aim of this study is to determine soil nematode fauna in the five different fruit orchards in Bilecik Province, Marmara Region with a holistic approach and to identify vermiform terrestrial nematodes up to the genus level. To date, *Anatonchus* Cobb, 1916, *Diphtherophora* de Man, 1880, *Funaria* Linde, 1938, *Telotylenchus* Siddiqi, 1960 and *Tripylella* Brzeski and Winiszewska-Slipinska, 1993 were not reported in any scientific report from Turkiye and apparently, this study is the first report of these genera regarding Turkiye’s nematofauna. Our contribution therefore is not only significantly expanding on what is known about nematode species in Türkiye, but also indicates available knowledge of the geographic records in the Marmara Region of Türkiye.

Nematodes as biological indicators provide valuable information about soil health. The study is conducted in five different fruit orchards habitats namely that of cherry, nectarine, olive, peach, plum and walnut. On the other hand, fruit tree orchards and their ecosystem are rarely discovered in terms of terrestrial nematofauna in Türkiye. Unfortunately, there is no study to our knowledge, related to free-living nematode biodiversity in the Marmara region. Regarding the total diversity, this study indicates a valuable contribution on the importance of faunistic studies of terrestrial nematode species.

### Species distribution and nematode abundance

The distribution of species in the fruit trees’ rhizosphere was investigated with an integrative approach to the species relative abundance and occurrence patterns. In fact, it is a difficult task to interpret the results of the distribution of soil nematodes. However, here we applied an interpretation scheme that consists of three frequency classes (low, medium, and high frequency) in terms of distribution patterns of nematodes in five different fruit types. According to these results, nematode genera that occurred in a high frequency at all fruit tree orchards are listed as follows: *Acrobeloides, Chiloplacus, Eucephalobus, Helicotylenchus, Malenchus, Panagrolaimus, Pratylenchus* and *Tylenchorhynchus.* These are cosmopolite nematode species and are distributed all over the world. Nematode genera that are occurring in a medium frequency at all fruit tree orchards are listed as follows: *Aphelenchoides, Aphelenchus, Aporcelaimellus, Paratylenchus, Tylenchus,* and *Xiphinema.* Finally, nematode genera that are occurring in a low frequency are listed as follows: *Alaimus, Amplimerlinus, Anatonchus, Aporcelaimus, Basiria, Belondira, Belonolaimus, Cephalobus, Cervidellus, Chromadorea, Clarcus, Criconema, Diplogaster, Dipterophera, Ecumenicus, Eudorylaimus, Eumonhystera, Filenchus, Funaria, Geomonhystera, Heterodera, Hoplolaimus, Labronema, Microdorylaimus, Monhystera, Mononchus, Mylonchulus, Nagelus, Nygolaimus, Plectus, Prionchulus, Prismatolaimus, Rhabditis, Rotylenchus, Telotylenchus, Teratocephalobus, Tobrilus, Tripylella, Tylocephalus* and *Steinernema.* Here, we need to place particular focus on the entomopathogenic nematode *Steinernema* spp., which occurred only in Plum and Olive orchards. Entomopathogenic nematodes are obligate parasites on insects in the soil and have a high potential to suppress many important pests, especially on fruit orchards, with the ability to survive for a long time under suitable conditions (Bedding et al., 1983; Kaya, 1985; Bedding, 1990).

### The nematode community

Comparing the nematode trophic groups within five fruit tree types showed a similar pattern. However, there are small differences that can be seen as a result of fruit tree characteristics. Apparently, bacterivorous nematodes were found to be the most common group in all the samples except in cherry trees. Plant parasitic nematodes were the most abundant trophic group in cherry orchards (69.1 %). Fungivorous nematodes have reached the highest percentage at nectarine orchards (16.7 %). On the other hand, in olive tree orchards, bacterivorous nematodes have reached up to 70.7 % of the total nematode community. Predator nematodes were found the most in plum tree orchards (47.3 %) as well as omnivore nematodes (6.8 %).

Additionally, the most diverse group in terms of species richness was within the order Dorylaimida. Omnivore nematodes as a persistent group in terms of soil monitoring, were found only in peach (6.8 %) and walnut (1.9 %) tree orchards with low percentages. The persistence of this group stems from their biology which also refers to an occurrence in mature and fertile soils and having a long-life cycle. The total percentage of omnivorous and predator nematodes has reached 54.1 % of peach tree orchards which shows clues of soil maturity and an undisturbed ecosystem in these areas. Walnut tree nematode assemblage was the most equalized composition in terms of feeding types. On the other hand, the abundance of bacterivorous, fungivorous, and plant parasitic nematodes had little variation between the sampling sites. This shows clues about the disturbance and agricultural practices’ pressure on the nematode community structure.

The balance of an ecosystem can be approached by the composition of nematodes in the soil. According to the colonizer-persister (cp) structure of the nematodes in these five fruit orchards, it is possible to say the nematodes associated with peach and walnut trees here showed the most stable environments in terms of soil nematode community structure. Schnürer et al. (1986) and Yeates (2007) mentioned that the most important factors affecting the nematode community are the environmental effects of regional and seasonal changes such as soil organic matter, texture, structure, chemical differences and moisture along with environmental disturbances caused by humans. Some studies that are conducted in agricultural ecosystems show a tendency of several patterns with respect to the seasonal fluctuations in the population dynamics of nematodes which have a short life cycle. Some authors noted significant annual density fluctuations (Fisher, 1968; Palomares-Rius et al., 2015), whereas another found no such distinct changes in nematode abundance (Strayer, 1985). Overall, the ecological indices and impact of environmental changes in terms of the nematode community allowed us to read the consistency of the fruit orchards in the surrounding soils of the Osmaneli district, in the Bilecik province, Marmara region, Türkiye. It is recommended to regularly monitor the nematode community and the soil properties for further explanation of this matter. Plant parasitic nematode suppression is a key element to plant protection. Here, once again we have seen the difference between a stable and disturbed soil nematode biodiversity, which may drastically affect the concept of intensive agricultural practices where soil disturbance is occurring and limit the balance of the diversity of soil microorganisms.

This last statement definitely discloses, once again, the critical position of terrestrial nematodes in the soil food web. The range of high tolerance may occur at different climatic conditions such as highly polluted habitats to mature soils which create habitats for tolerant species and sensitive species. Nematodes have low mobility and rapid responses to disturbance and enrichment changes. Life-cycle properties of nematodes ranging from 6 days to over 2 years provide wide opportunities, perspectives, and practical tools to scientists not only for understanding environmental changes but also conservation of soil biodiversity.

Finally, our study of the fauna of terrestrial nematodes at five fruit orchards of the Bilecik Province might give a beneficial contribution on the monitoring of terrestrial nematode fauna of Türkiye and show how nematodes can be useful for soil monitoring as a rising interest.
